# Longitudinal microstructural changes of cerebral white matter and their association with mobility performance in older persons

**DOI:** 10.1371/journal.pone.0194051

**Published:** 2018-03-19

**Authors:** Nicola Moscufo, Dorothy B. Wakefield, Dominik S. Meier, Michele Cavallari, Charles R. G. Guttmann, William B. White, Leslie Wolfson

**Affiliations:** 1 Center for Neurological Imaging, Department of Radiology, Brigham and Women’s Hospital, Harvard Medical School, Boston, Massachusetts, United States of America; 2 Department of Neurology, University of Connecticut School of Medicine, Farmington, Connecticut, United States of America; 3 Division of Hypertension and Clinical Pharmacology, Calhoun Cardiology Center (WBW), University of Connecticut School of Medicine, Farmington, Connecticut, United States of America; University of California, San Francisco, UNITED STATES

## Abstract

Mobility impairment in older persons is associated with brain white matter hyperintensities (WMH), a common finding in magnetic resonance images and one established imaging biomarker of small vessel disease. The contribution of possible microstructural abnormalities within normal-appearing white matter (NAWM) to mobility, however, remains unclear. We used diffusion tensor imaging (DTI) measures, i.e. fractional anisotropy (FA), mean diffusivity (MD), axial diffusivity (AD), radial diffusivity (RD), to assess microstructural changes within supratentorial NAWM and WMH sub-compartments, and to investigate their association with changes in mobility performance, i.e. Tinetti assessment and the 2.5-meters walk time test. We analyzed baseline (N = 86, age ≥75 years) and 4-year (N = 41) follow-up data. Results from cross-sectional analysis on baseline data showed significant correlation between WMH volume and NAWM-FA (r = -0.33, p = 0.002), NAWM-AD (r = 0.32, p = 0.003) and NAWM-RD (r = 0.39, p = 0.0002). Our longitudinal analysis showed that after 4-years, FA and AD decreased and RD increased within NAWM. In regional tract-based analysis decrease in NAWM-FA and increase in NAWM-RD within the genu of the corpus callosum correlated with slower walk time independent of age, gender and WMH burden. In conclusion, global DTI indices of microstructural integrity indicate that significant changes occur in the supratentorial NAWM over four years. The observed changes likely reflect white matter deterioration resulting from aging as well as accrual of cerebrovascular injury associated with small vessel disease. The observed association between mobility scores and regional measures of NAWM microstructural integrity within the corpus callosum suggests that subtle changes within this structure may contribute to mobility impairment.

## Introduction

Magnetic resonance images (MRI) of the brain from elderly individuals frequently show areas of white matter damage, characterized by a T2 hyperintense signal and therefore termed white matter hyperintensities (WMH). Most often these lesions extend outward from the anterior and posterior horns of the lateral ventricles although other hemispheric locations including subcortical regions are observed. Longitudinal studies indicate that WMH increase mostly through expansion of existing WMH, rather than newly forming lesions [1]. A multimodal MRI study provided evidence of damage extending from WMH into larger adjacent areas of normal-appearing white matter (NAWM) characterized by reduced blood flow [2] thus suggesting an underlying ischemic mechanism of WMH expansion. This explanation is supported by epidemiological studies showing associations between WMH and vascular disease risk factors, including hypertension [3–5], diabetes [6] and obesity [7].

The location and extent of WMH have been linked to cognitive impairment, depression and urinary dysfunction [8–11]. Additionally, cross-sectional and longitudinal MRI studies using observation-based assessment or quantitative evaluation of brain white matter damage have established a link between WMH volume and location and measures of mobility [12–18]. The predisposition of WMH to occur in frontal areas adjacent to the lateral ventricles likely explains their deleterious effect on mobility, cognition (primarily executive function) and urinary control [10, 12, 17].

While the impact of WMH burden on mobility function is well established and supported by several studies, the presence of abnormalities within the NAWM and their contribution to mobility is unclear. Changes in NAWM using diffusion tensor imaging (DTI) have been extensively studied in relation to aging and found to be associated with functional decline [19–23]. DTI is an MR image acquisition technique that is sensitive to water diffusion within brain tissue and can provide biologically relevant information on its microstructure. Axial diffusivity (AD, parallel diffusion) and radial diffusivity (RD, perpendicular diffusion) are two DTI indices that measure diffusion of water along or perpendicular to white matter tracts, respectively. Fractional anisotropy (FA) represents a normalized measure of the directionality of water diffusion and relates to both AD and RD. These diffusion indices are considered to reflect the microstructural integrity of the tissue analyzed [24]. FA tends to be higher in areas dense with uniformly oriented tracts of myelinated axons and it is lower when the myelin sheath is thinner or damaged, and/or axons are sparse. DTI has been used to study the relationship between brain WM microstructural integrity and mobility measures [13, 15, 20, 25, 26]. To our knowledge, only four cross-sectional studies have previously focused on NAWM abnormalities and their relationship to mobility. These studies suggest that reduced microstructural integrity within NAWM may play a role in the development of gait disturbances in older persons [13, 20, 27, 28]. A recent report describing the results of a five-year longitudinal study also supports a role of cerebral white matter microstructural integrity in mobility decline [29].

The aims of this work in which we analyzed elderly subjects at baseline (2005–2006) and after four years (2009–2010) were: 1) to characterize longitudinal DTI changes in the cerebral white matter (i.e. NAWM and WMH) and 2) to explore their relation to changes in mobility performance. We measured diffusion indices within both the NAWM and WMH to understand and compare the microstructural substrates and evolution of white matter damage. We hypothesized that not only macroscopic white matter damage, in the form of WMH, but also microstructural abnormalities of the NAWM, as measured by diffusion MRI, may be linked to the development of mobility impairment. To test this hypothesis, we analyzed the association between mobility performance and DTI measures of damage to selected white matter tracts. For this explorative analysis tract selection was based on our previous findings of significant association between regional WMH and mobility impairment [1, 30]. Since, to our knowledge, this is one of the first longitudinal MRI studies utilizing quantitative DTI to assess changes in brain microstructural integrity and their relationship with mobility changes, we complemented the hypothesis-driven regional analysis of selected white matter tracts with assumption-free voxel-based approach to assess diffusion changes in the whole brain.

## Methods

### Subjects

The Institutional Review Board (IRB) of both the University of Connecticut Health Center (Human Subjects Protection Office) and Brigham and Women's Hospital (Partners Human Research Committee) approved the study protocol.

The sample in the present analysis is a cohort of a completed observational study. The details of the parent study have been previously reported [20, 31]. Briefly, 99 community residing subjects, aged 75–89 years, were enrolled and stratified by age and mobility function using the Short Physical Performance Battery [32]. Exclusion criteria included: evidence of neurologic and medical conditions known to impair mobility, compromised cognition (Mini-Mental Status Exam, MMSE < 24) [33], inability to undergo an MRI or walk 10 meters independently in less than 50 seconds [31]. After providing a written informed consent, all subjects underwent physical, neurological and cognitive assessments as well as brain MRI. The current report consists of a subset of 86 subjects who completed the full MRI examination, including DTI at baseline. Of those, 41 subjects also completed the 4-year follow-up assessments and were included in the longitudinal analyses. Of the 45 subjects who had baseline data but did not have 4-year data available, 23 were lost to follow-up, and MRI data for the other 22 subjects did not include DTI data. Imaging and clinical raters were blinded to clinical mobility and imaging outcomes, respectively.

### Mobility assessment

Study subjects underwent mobility assessment that included several tests as previously described [17]. For the present analysis we used two established measures of mobility performance, the 2.5-meter walk-time [32] and the composite Tinetti mobility assessment score [34], which showed longitudinal changes over the 4-year follow-up period as well as significant associations with WMH in previous analysis [17]. The Tinetti score is designed to assess the balance and gait of elderly adults. It consists of nine individually scored tasks testing balance during: sitting, standing, postural transitions, turning and stressed conditions. It also has seven measures of gait quality: initiation, path, step length, step symmetry, step continuity, truncal control and stance. A score below 19 (sum of the individual scores) indicates high fall risk and a score below 24 indicates some fall risk.

### MRI acquisition

MR imaging of the brain was performed using a 3T Siemens Allegra scanner (Siemens, Erlangen, Germany). Structural series for anatomical co-registration and segmentation included T1-weighted Magnetization Prepared Rapid Gradient Echo (MPRAGE, 176 1-mm thick axial slices, TR/TE = 2500/2.74 msec, TI = 900 msec, matrix size = 256×208, in-plane pixel spacing = 1×1 mm); 3D-Fast Spin Echo (T2, 176 1-mm thick sagittal slices, TR/TE = 2500/353 msec, matrix size = 256×220, in-plane pixel spacing = 1×1 mm), and Fluid Attenuated Inversion Recovery (FLAIR, 128 1.3-mm thick sagittal slices, TR/TE = 6000/353 msec, TI = 2200 msec, matrix size = 256×208, in-plane pixel spacing = 1×1 mm). Diffusion Tensor Imaging (DTI) was performed using a standard twice-refocused EPI sequence with TR/TE = 5800/87 msec, FOV = 20 cm, acquisition and reconstruction matrices = 128×96 and 128×128, diffusion sensitizing orientations in twelve directions with one B0, and eight averages for each direction, b = 1000 s/mm^2^, 45 contiguous axial slices of 3 mm section thickness. Structural series were corrected for magnetic field-related signal inhomogeneity [35] and the FLAIR and T2 series were registered (linear affine) to the MPRAGE series [36]

### DTI analyses of supratentorial white matter

#### DTI indices within NAWM and WMH

**Fig 1:**
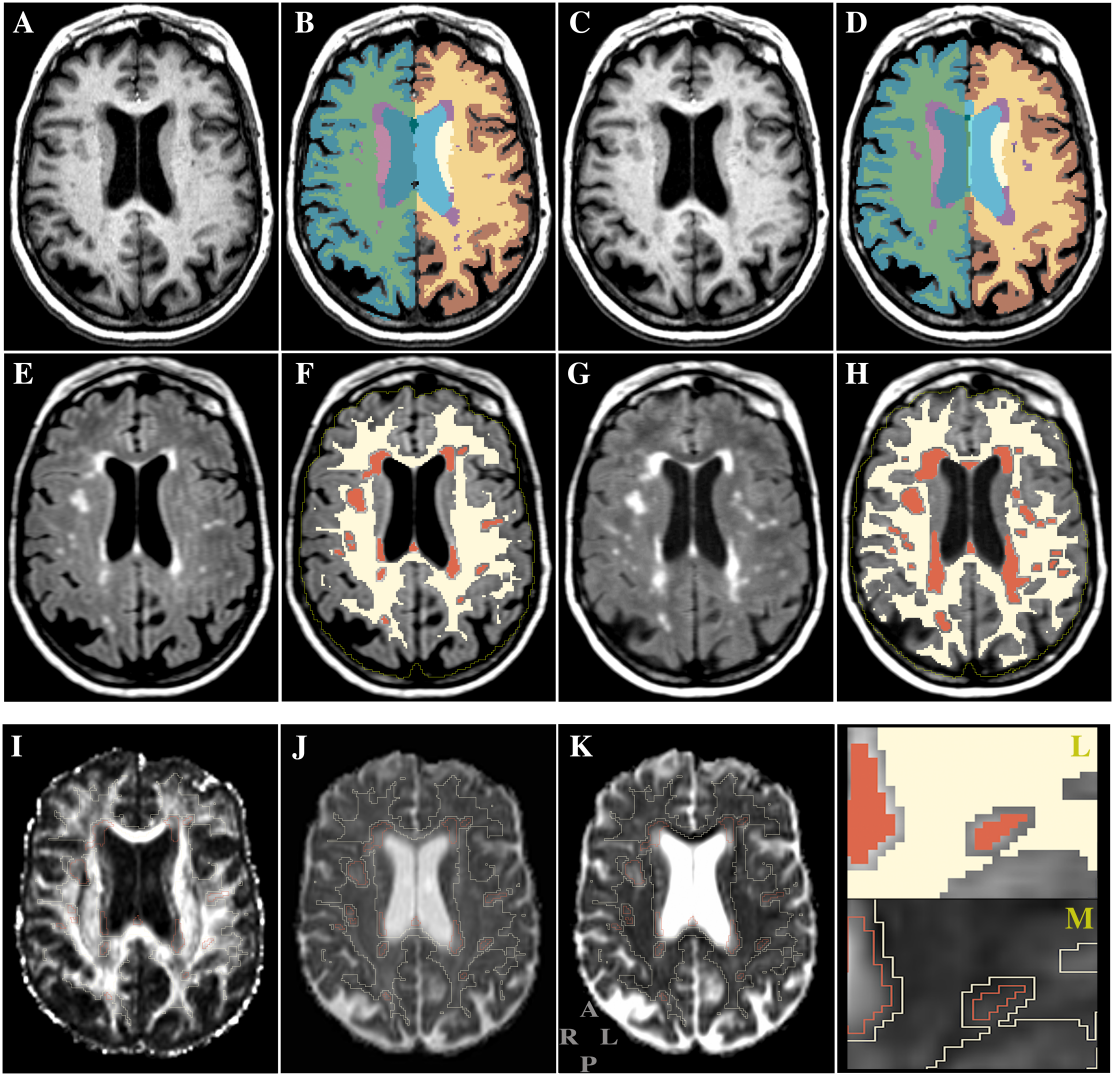
Example of structural and diffusion MR images from the study. Panels show the same brain region in axial view at baseline (A-B, E-F, I-K) and at 4-years (C-D, G-H). *Top row*: T1-weighted MPRAGE images. *Middle row*: T2-weighted FLAIR images. Panels B and D show the brain tissue segmentation maps (parc) obtained with FreeSurfer [37]. The parc WM and the WMH maps were merged as shown in F (baseline) and H (4 years) where NAWM (eroded) is indicated in light yellow and WMH in red. We used these (i.e. F and H) as masks to measure the diffusion indices within the NAWM and WMH compartments. The yellow line in panels F and H represents the outline of the intracranial cavity. *Bottom row*: panels show an example of diffusion maps from the baseline time point. The outline-only of NAWM and WMH map in panel F is overlaid on the FA (I), AD (J) and RD (K) images. The two small panels at bottom right show a close view of a section of the overlaid mask as filled (L, from panel F) and outlined (M, from panel K). Standard radiological orientation is shown at bottom left of panel K with A = anterior; R = right; L = left; P = posterior.

The areas of microvascular damage observed as white matter hyperintensities (WMH) in FLAIR images and the intracranial cavity were identified as previously described [30]. For each subject the anatomical brain parcellation map (parc, Fig 1, panels B and D) was also obtained using the segmentation function *recon-all*, part of the FreeSurfer software [37]. For each subject the WM in the parc and WMH maps above were consolidated into one single map with NAWM and WMH distinctly labeled (Fig 1, panels F and H). This map was then used in the subsequent steps as mask for regional assessment of DTI metrics and for quantitative volumetric assessments. To account for differences in head size we expressed the WMH volumes (WMHv) as percent of the intracranial cavity volume (ICV, example of outline in Fig 1, panels F and H). Since infratentorial WMHv were virtually absent in the study sample we focused our analysis on the supratentorial white matter by excluding the brain areas in the parc map identified as brainstem and cerebellar white matter by the FreeSurfer segmentation method [37]. We defined NAWM as the cerebral white matter voxels not classified as WMH by our method.

DTI data were processed using the FSL-FDT software standard methodology (www.fmrib.ox.ac.uk/fsl), which included motion and eddy-current correction steps, to derive the fractional anisotropy (FA), mean diffusivity (MD), axial diffusivity (AD), and radial diffusivity (RD) maps and the pre-gradient T2-weighted S0 set. While we used the MD maps in some cross-sectional analysis and in the voxel-based image processing steps within SPM 8 (see below) the study focused on FA, AD and RD maps. In order to align the diffusion maps to the structural MPRAGE images the S0 was registered with an affine transform to the T2-weighted images previously affine-registered to the MPRAGE series. The resulting transformation matrix was then applied to the FA, AD and RD maps using the *sinc* function interpolation. Thus for each subject all relevant images, i.e. structural, diffusion, brain segmentation maps, including WMH and NAWM maps, were co-aligned in the T1-weighted MPRAGE space. To minimize partial volume effect caused by inclusion of non-NAWM voxels near borders with gray matter, cortical-spinal fluid and WMH areas, we performed an 8-neighbor 'square' erosion step (Matlab, Mathworks Inc., Natick, Massachusetts) on peripheral voxels of the supratentorial NAWM. An example of the eroded map output is shown in Fig 1 (panels F and H, and bottom right panel). Next for each subject we used the eroded-NAWM and WMH masks to extract and calculate DTI coefficients within these two supratentorial white matter tissue classes (example in Fig 1, panels I, J, K).

#### DTI indices within selected tract-based regions of interest

**Fig 2:**
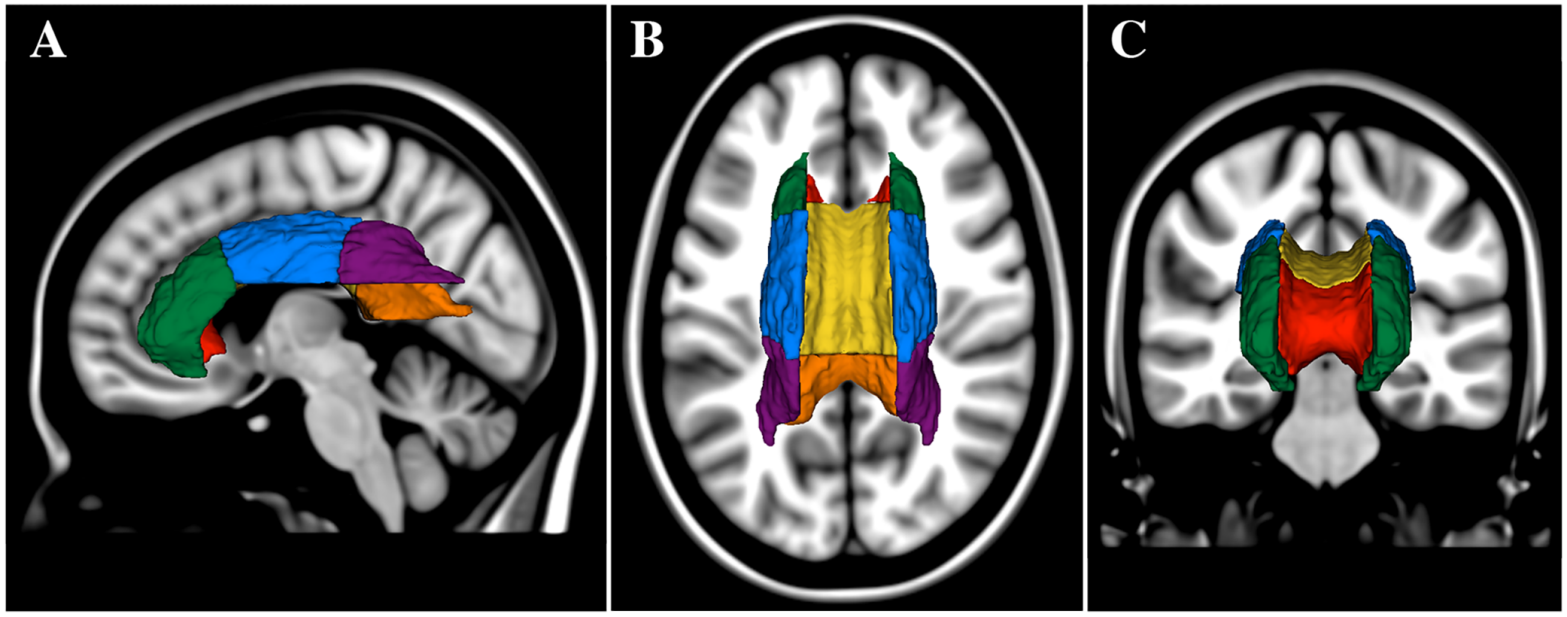
Relative spatial location of the regions of interest analyzed. Three-dimensional models of the sub-regions of the corpus callosum and corona radiata are illustrated. Models were produced in Slicer 3D (slicer.org) using the MNI white matter parcellation map and they are shown overlaid on the MNI brain in sagittal (A), axial (B) and coronal (C) views. Regions shown are the anterior corona radiata (ACR, green), superior corona radiata (SCR, blue), posterior corona radiata (PCR, purple), genu of corpus callosum (GCC, red), body of corpus callosum (BCC, yellow), and splenium of corpus callosum (SCC, orange). Views are from left (A), top (B) and front (C) of head. For the regional assessment of the diffusion indices we used a mask with skeletonized ROIs in the subject native space.

For the tract-based region-of-interest (ROI) analysis we followed the standard Tract Based Spatial Statistics procedure in FSL [38]. We obtained the skeleton map of the WM (FA threshold ≥ 0.2) and the corresponding regional WM parcellation map derived from the John Hopkins University WM label map (JHU-wmparc) as part of FSL-FDT software package. Next we performed a de-projection step to move the ROIs map to the native space of each subject and obtained unique tract-based maps that reflected each study participants' anatomy. We then used this subject-specific ROI mask to extract the FA, AD and RD in the genu, body and splenium of the corpus callosum (GCC, BCC and SCC, respectively), and the anterior, superior and posterior parts of the corona radiata (ACR, SCR and PCR, respectively). Their relative anatomical spatial location is illustrated in Fig 2.

The selection of ROIs was based on our previous studies [1, 30] that showed associations between mobility performance and regional WMH burden in those areas, which are crossed by anatomically important inter-hemispheric and projection neural tracts. In order to measure the DTI indices of NAWM-only within each region, for each subject we combined the eroded-NAWM and the ROI masks of the tracts as defined by the JHU-wmparc. Only those pixels that were within both the selected ROIs and NAWM masks were included. Diffusion indices were expressed as mean value (sum of the pixel values divided by the number of pixels) in arbitrary units (FA) or μm^2^/sec (AD, RD).

### Voxel-based whole brain analysis

To expand our understanding of the localization of brain microstructural abnormalities potentially involved in the development of mobility impairment, we investigated the association between 4-year DTI changes and mobility changes using a whole-brain voxel-based approach [39] where both gray and white matter were included. Subjects' FA, MD, AD and RD maps were processed using the Statistical Parametric Mapping (SPM 8) software package (http://www.fil.ion.ucl.ac.uk/spm). All the DTI diffusion maps were first resampled to the EPI template brain. The MD and FA maps from both time points, i.e. baseline (TP1) and 4-year follow-up (TP2), were iteratively (x6) spatially normalized with a nonlinear function to the average MD and FA maps derived from all individual MD and FA diffusion maps, respectively. To improve alignment, FA was used in alternate combination with MD during the normalization routine. The spatial transforms were then applied to AD and RD maps. For each subject the differential diffusion FA, AD and RD maps were obtained by subtraction of the co-registered images at the two time-points (TP2—TP1). The differential diffusion maps were then smoothed using a Gaussian kernel of 6 mm FWHM (Full Width at Half Maximum). In this analysis the subjects' DTI images were in standard Montreal Neurological Institute (MNI) space. We then performed a voxel-wise general linear regression model analysis using the smoothed images and walk time or Tinetti mobility change measures. The changes in mobility performance were expressed as percent difference relative to TP1 (mobility change = 100*[TP2 score—TP1 score]/TP1 score).

### Statistical analysis

We assessed the strength of the association between WMHv and DTI indices within NAWM and WMH at baseline (N = 86) using Spearman correlation. DTI differences between NAWM and WMH areas were assessed with the Wilcoxon signed rank test.

We assessed the significance of the change over time in the DTI data (AD, FA, RD) and in the two mobility measures, i.e. Tinetti total mobility assessment score and Log_10_ transformed 2.5-meter walk-time, with simple paired analyses among the 41 subjects with both baseline and 4-year data using Wilcoxon signed rank test.

In our final multivariate models we used all available data at baseline (N = 86) and at four years (N = 41) to evaluate the association of DTI indices with mobility measures over time. Each linear mixed model included one mobility measure (dependent variable), one DTI measure (independent variable) and age, BMI, baseline WMHv, and gender as covariates. We used SAS version 9.4 (SAS Institute, Inc., Cary, NC) and R (https://www.r-project.org) for the analyses. The threshold for statistical significance was α ≤0.05 (two-tailed). We used the false discovery rate (FDR) procedure [40] to correct for multiple tests involving the ROIs (N = 18) and the mobility variable. Due to the exploratory nature of this study (identify candidate ROI for further analysis), we chose an FDR of 30% (Q = 0.3) and thus accepted that approximately one-third of observed significant associations falling below the corresponding FDR critical p-value of significance = Q*(rank/N)) could be subject to type I error (false positive). This yields significance thresholds for the top three associations of p≤0.017 (0.30*(1/18)), p≤0.033 (0.30*(2/18)), and p≤0.050 (0.30*(3/18)), respectively. For voxel-wise analysis (SPM 8) we used a general linear model controlling for age baseline WMHv, and gender. Threshold for significance was set to p≤0.05 at the voxel level after correction for multiple comparisons within each cluster.

## Results

### Demographics and sample characteristics

Characteristics of the study subjects are presented in Table 1. At baseline the mean age for the sample (N = 86) was 82.9 ± 3.9 years and 55% were female. Subjects were non-Hispanic whites; 93% completed high school or a higher educational level. Forty-one subjects completed the 4-year follow-up assessments (mean age at completion was 85.9 ± 4.1 years and 54% female) and had DTI data. Baseline characteristics of the 41 completers (age, BMI, gender, education) were similar to the 45 non-completers with exception of age. The non-completers were on average two years older (84 vs. 82 years, p = 0.02). Baseline DTI measures and WMH volumes of the dropouts were similar to the completers (p>0.05). All participants entered the study without dementia, with a Mini Mental State Examination (MMSE) score > 24.

**Table 1:** Characteristics of study subjects at baseline.

Sample (n)	86
Age, mean (SD)	82.90 (3.92)
Female, n (%)	47 (54.65)
BMI, mean (SD)	26.31 (4.15)
Education, n (%)	
< HS	6 (6.98)
HS Graduate	30 (34.88)
College Graduate	27 (31.40)
Post Graduate	23 (26.74)
Mini-mental Status Exam	28.45 (1.38)
**Mobility**	
Tinetti mobility assessment	25.81 (2.62)
8-ft Walk Time (s)	3.17 (0.78)

In the completers-only group (N = 41) the average MMSE decreased slightly from 28.56 to 28.15 (p = 0.165) over the 4 years of the follow-up. We observed a significant although relatively modest decrease in mobility over 4 years. The Tinetti mobility assessment score decreased from 26.39 to 24.37 (p<0.01) moving the mean subject score from the low to medium fall risk group, while the average 2.5-meter walk-time increased from 3.03 to 3.39 seconds (p = 0.04) (Table 2).

**Table 2:** Characteristics of completers-only subjects.

	Baseline	4 years	p-value*
Sample (n)	41	41	
Age, mean (SD)	81.85 (4.10)	85.90 (4.13)	
Female, n (%)	22 (53.66)		
BMI, mean (SD)	25.78 (4.87)	25.49 (4.77)	0.26
Education, n (%)			
< HS	1 (2.44)		
HS Graduate	13 (31.71)		
College Graduate	13 (31.71)		
Post Graduate	14 (34.15)		
Mini-Mental Status Exam	28.56 (1.41)	28.15 (1.63)	0.16
**Mobility**			
Tinetti mobility assessment	26.39 (2.28)	24.37 (3.26)	<0.01
8-ft Walk Time (s)	3.03 (0.81)	3.39 (1.39)	0.04

* Wilcoxon signed rank test

### Baseline analysis of DTI indices in NAWM and WMH

**Fig 3:**
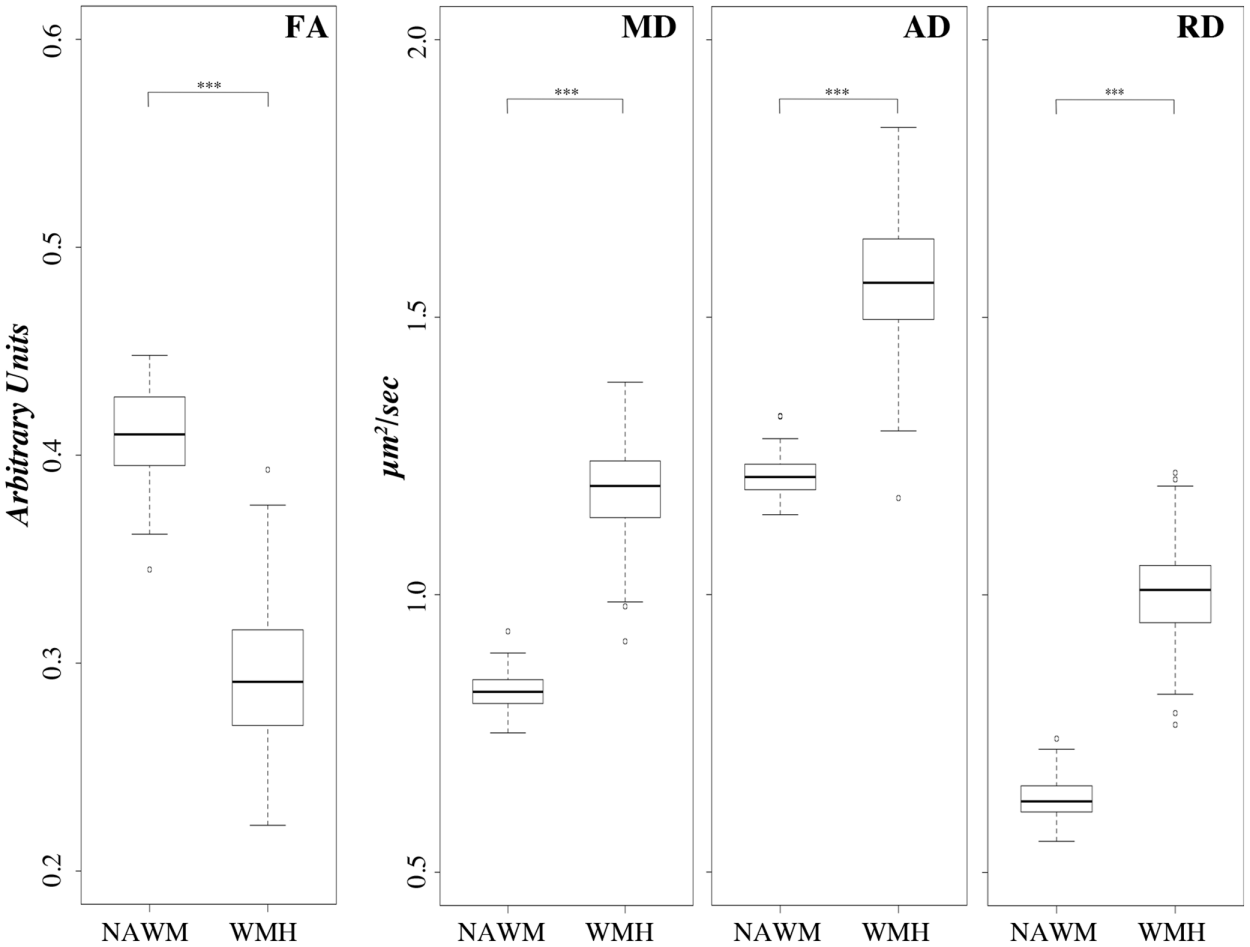
Comparison of DTI indices between NAWM and WMH at baseline. Boxplots illustrate the values distribution (y-axis) of the fractional anisotropy (FA), mean diffusivity (MD), axial diffusivity (AD) and radial diffusivity (RD) indices within the normal-appearing white matter (NAWM) and within the white matter hyperintensity (WMH) areas. FA expressed in arbitrary units; MD, AD and RD expressed as μm^2^/sec. Baseline sample, N = 86. *** Statistical significance of p≤10^−7^ in Wilcoxon signed rank one-sample test.

Cross-sectional comparison of diffusion indices between NAWM and WMH on the baseline sample (N = 86) showed that the global NAWM-FA was about 40% higher than WMH-FA (0.41 ± 0.02 vs 0.29 ± 0.03 arbitrary units, p<0.01), NAWM-AD was 23% lower than WMH-AD (1.21 ± 0.03 vs 1.57 ± 0.11 μm^2^/sec, p<0.01) and NAWM-RD was 37% lower than WMH-RD (0.63 ± 0.04 vs 1.00 ± 0.09 μm^2^/sec, p<0.01). These results are shown in Fig 3, which includes also MD for completeness. As expected, MD values fall between those of AD and RD for both NAWM and WMH. Differences in DTI indices between NAWM and WMH are highly significant with WMH values clearly indicating higher local water diffusivity and lower anisotropy compared to NAWM. In a correlation analysis the NAWM indices at baseline were not associated with mobility measures after controlling for age, gender and baseline WMHv (S1 Table).

### Baseline association between DTI indices and WMH volume (WMHv)

**Fig 4:**
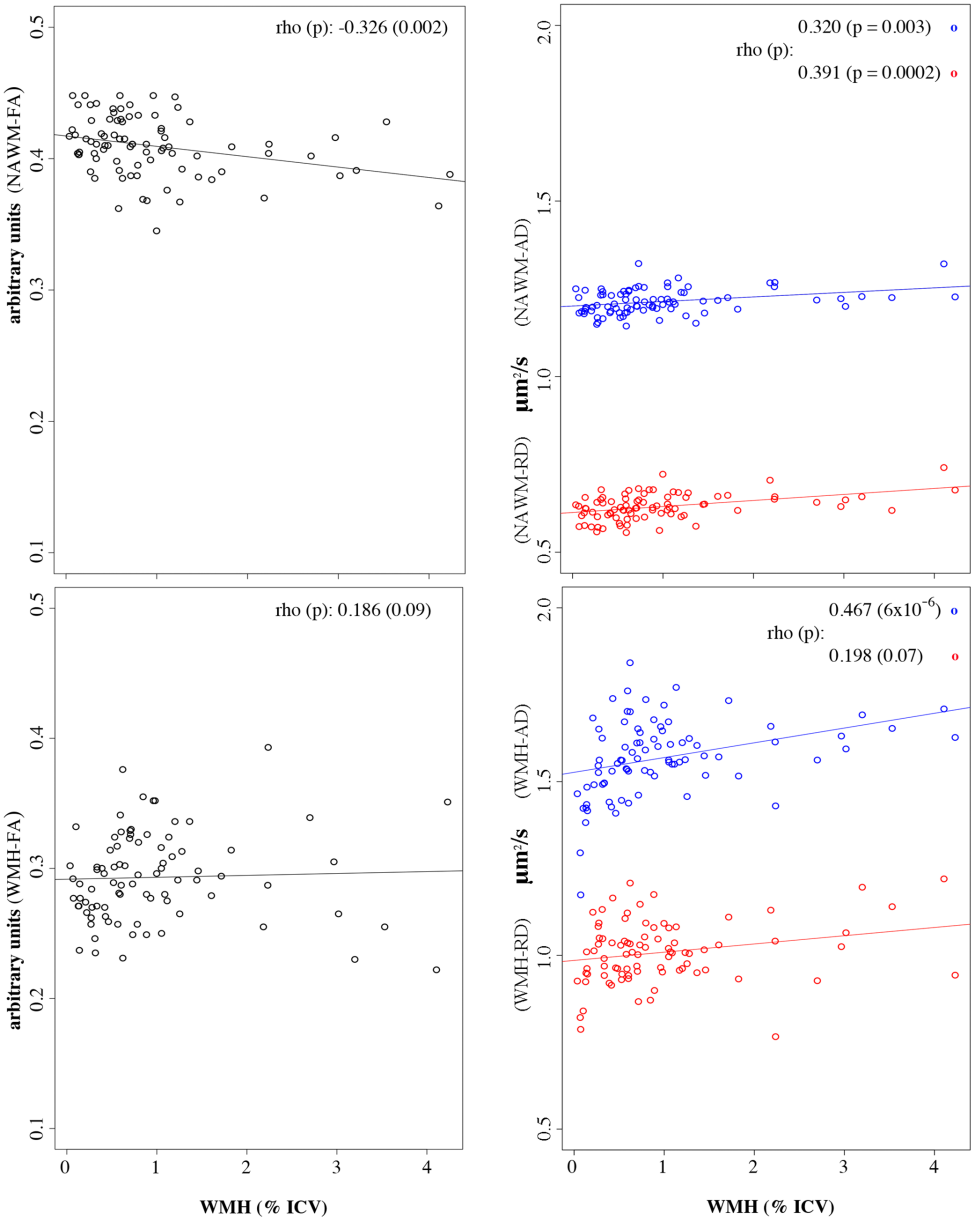
WMHv burden and DTI indices within NAWM and WMH. Figure shows results of Spearman correlations (rho) between WMH volume (WMHv) and DTI indices within areas of NAWM (top panels) and WMH (bottom panels) on baseline data. The x-axes represent WMHv expressed as percent of the intracranial cavity volume (ICV). The y-axes represent the DTI indices, namely the FA (left two panels, black circles) and AD and RD (right two panels, blue and red circles, respectively). Circles represent the average value within supratentorial NAWM or WMH areas for each study subject. Correlation remained significant after controlling for age and gender. Lines represent a fitted linear regression trendline.

We found significant association between WMHv and DTI indices within the NAWM in the larger baseline sample (N = 86, Fig 4). Subjects with larger WMH burden were more likely to show lower NAWM-FA (r = -0.33, p = 0.002) and both higher NAWM-AD (r = 0.32, p = 0.003) and NAWM-RD (r = 0.39, p = 0.0002). Lesion volume (WMHv) was significantly associated with WMH-AD (r = 0.47, p<10^−5^) but not with WMH-FA or WMH-RD (p>0.05). Correlations remained significant after controlling for age and gender.

### Longitudinal DTI changes (paired analysis)

**Fig 5:**
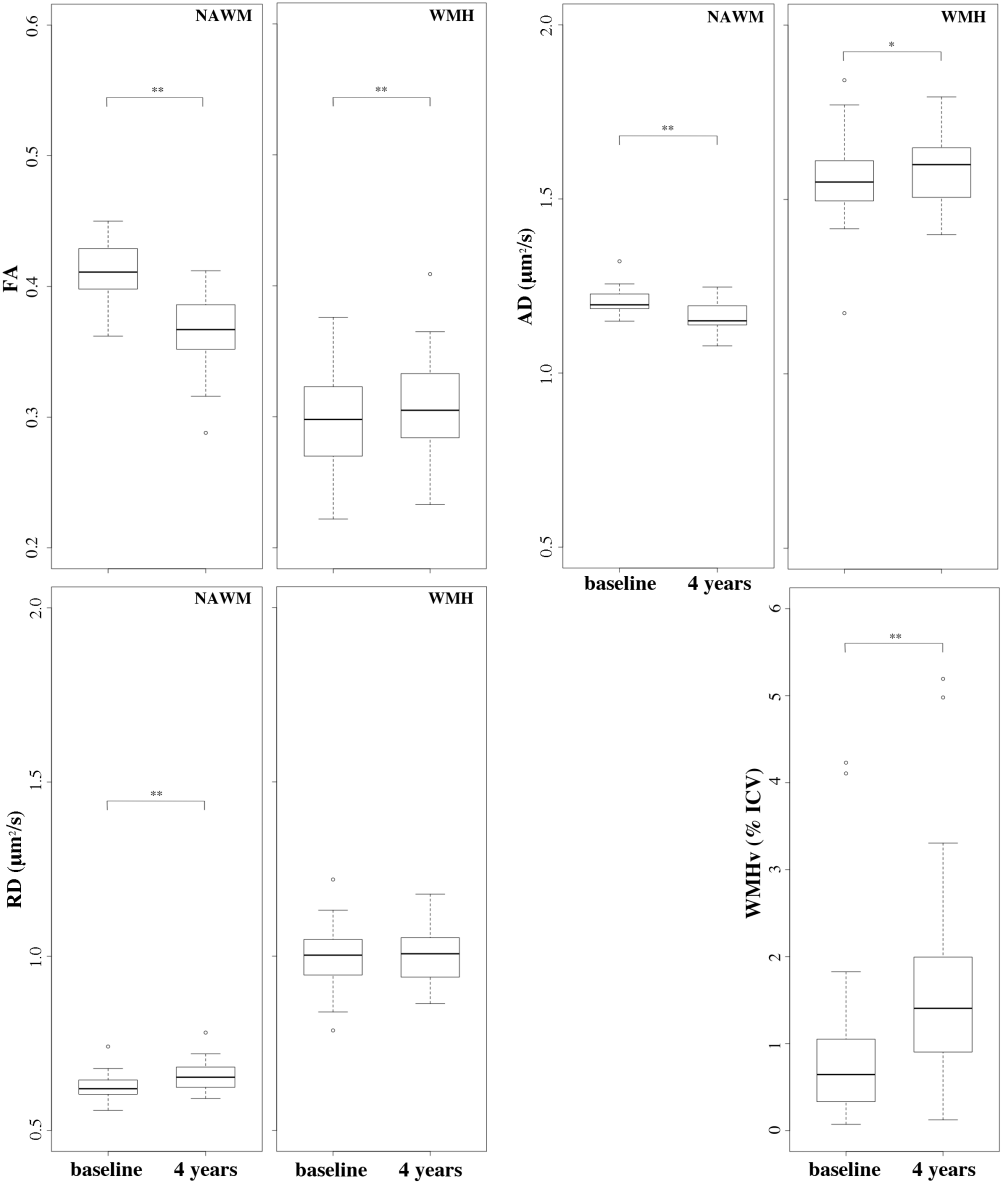
Baseline and 4-year FA, AD and RD indices in supratentorial WM. Boxplots illustrate the DTI indices values distribution (y-axis) at baseline and at 4-years (x-axes) in the completers-only sample (N = 41). *Top left*: fractional anisotropy (FA, arbitrary units) within normal-appearing white matter (NAWM) and WMH for comparison. *Top right*: axial diffusivity (AD, μm^2^/sec) within NAWM and WMH. *Bottom left*: radial diffusivity (RD, μm^2^/sec) within NAWM and WMH. *Bottom right*: white matter hyperintensity volume (WMHv, as percent of the intracranial cavity volume, i.e. % ICV). Statistical significance assessed with Wilcoxon signed rank paired sample test: * p<0.05; ** p<10^−3^.

Results of 4-year changes in the completers-only sample (N = 41) are reported in Fig 5 (see also S1 Scatterplot). Over 4 years, NAMW-FA and NAWM-AD decreased on average by about 10% and by 3% (p<0.01), respectively, while NAWM-RD increased by 5% (p<0.01). Over the same time interval, WMH-FA and WMH-AD increased by about 7% (p<0.01) and 2% (p = 0.03), respectively, while WMH-RD did not change (p = 0.94). The differences in FA, AD and RD between NAWM and WMH were significant at both baseline and 4 years (p = 0.01). Total lesion volume (i.e. WMHv) increased on average by 82% over the 4 years (0.99±0.99 at baseline vs 1.80±1.33 at follow-up, p<0.01) (Fig 5).

The 4-year DTI values of tract-specific NAWM in the completers-only sample are shown in Table 3. We observed significant decrease of FA and increase of RD in the genu and body of corpus callosum (p<0.01), i.e. GCC-NAWM and BCC-NAWM. We also found a significant increase of AD within GCC-NAWM. Significant differences at follow-up were found also in the NAWM within the corona radiata, namely an increase in the AD of the superior part (SCR) and a decrease in the RD of the posterior part (PCR) (Table 3).

**Table 3:** Longitudinal changes of regional DTI indices.

DTI Data	Baseline mean (SD)	4 year mean (SD)	Change (↑↓)	P-value
**FA**				
BCC-NAWM	0.59 (0.04)	0.56 (0.04)	↓	**<0.01**
GCC-NAWM	0.65 (0.05)	0.63 (0.05)	↓	**<0.01**
SCC-NAWM	0.75 (0.03)	0.75 (0.04)	=	0.99
ACR-NAWM	0.42 (0.04)	0.43 (0.04)	=	0.20
PCR-NAWM	0.49 (0.04)	0.50 (0.06)	=	0.15
SCR-NAWM	0.48 (0.05)	0.48 (0.04)	=	0.28
**AD**				
BCC-NAWM	1.63 (0.05)	1.62 (0.05)	=	0.15
GCC-NAWM	1.63 (0.08)	1.68 (0.08)	↑	**<0.01**
SCC-NAWM	1.59 (0.05)	1.59 (0.10)	=	0.08
ACR-NAWM	1.25 (0.05)	1.24 (0.05)	=	0.46
PCR-NAWM	1.35 (0.05)	1.33 (0.09)	=	0.40
SCR-NAWM	1.25 (0.07)	1.25 (0.07)	↑	**0.008**
**RD**				
BCC-NAWM	0.55 (0.06)	0.59 (0.06)	↑	**<0.01**
GCC-NAWM	0.49 (0.07)	0.52 (0.09)	↑	**<0.01**
SCC-NAWM	0.33 (0.05)	0.33 (0.06)	=	0.63
ACR-NAWM	0.62 (0.05)	0.62 (0.06)	=	0.52
PCR-NAWM	0.59 (0.05)	0.57 (0.07)	↓	**0.02**
SCR-NAWM	0.55 (0.04)	0.55 (0.04)	=	0.82

N = 41. Significance (p-value) assessed with Wilcoxon signed rank test. FA expressed in arbitrary units; AD and RD expressed in μm^2^/sec.

### Multivariate longitudinal analysis: Mobility and DTI changes

#### Mobility and DTI changes in global NAWM

We analyzed the relationship between changes in mobility and changes in the microstructural integrity of NAWM over the 4-year follow-up using a linear mixed model analysis controlling for age, gender, BMI and baseline WMHv (Table 4). Besides the marginally significant correlation between NAWM-AD and Tinetti score (p = 0.06), we observed no significant association between changes in the global diffusion indices of NAWM and Walk Time or Tinetti score. Changes in WMH-AD correlated with Tinetti score (p = 0.04). White matter lesion burden (WMHv), was significantly associated with both walk time and Tinetti Mobility Assessment (p<0.01) independent of age and gender.

**Table 4:** Longitudinal analysis of diffusion indices within NAWM and WMH and mobility: Linear mixed model estimates and standard errors.

DTI	Walk Time*		Tinetti Mobility	
	Beta (SE)	P-Value	Beta (SE)	P-Value
NAWM-FA	-0.52 (0.95)	0.59	-16.44 (10.98)	0.14
WMH-FA	0.61 (0.63)	0.33	-5.25 (7.22)	0.47
NAWM-AD	0.87 (0.65)	0.19	-14.65 (7.66)	0.06
WMH-AD	0.16 (0.23)	0.49	-5.60 (2.63)	**0.04**
NAWM-RD	0.95 (0.69)	0.17	-0.57 (8.05)	0.94
WMH-RD	-0.01 (0.25)	0.96	-2.92 (2.92)	0.32
**WMH (Volume)**				
WMHv*	0.05 (0.02)	**<0.01**	-0.66 (0.23)	**<0.01**

* Values were log-transformed (log_10_).

AD: axial diffusivity; FA: fractional anisotropy; RD: radial diffusivity; NAWM: normal-appearing cerebral white matter. All models controlled for age, gender, BMI, time point and WMHv (log_10_).

#### Mobility and DTI changes in tract-specific NAWM

Table 5 illustrates how the relative amount of NAWM in each tract-specific region that we analyzed changed in the completers-only sample (N = 41). As previously reported the corona radiata is proportionally more affected by WMHv than the corpus callosum [30] and within these two tracts the posterior (PCR) and anterior (GCC) parts are the two sub-regions with most WMHv. Thus the percent regional amount of NAWM in decreasing order is SCC > BCC > GCC >SCR > ACR > PCR.

**Table 5:** Regional NAWM as percent of the ROI volume.

	BASELINE	4-YEARS
**GCC-NAWM**	94.8 ± 6.8 (97.1, 62.2–100)	88.2 ± 11.3 (92.1, 44.3–99.1)
**BCC-NAWM**	97.1 ± 5.9 (99.1, 64.0–100)	94.3 ± 6.6 (94.9, 60.5–99.9)
**SCC-NAWM**	98.2 ± 3.1 (99.5, 86.4–100)	94.6 ± 5.3 (96.8, 81.1–99.9)
**ACR-NAWM**	87.8 ± 15.3 (92.3, 13.0–99.9)	77.6 ± 19.2 (82.9, 17.5–97.7)
**SCR-NAWM**	93.1 ± 13.8 (98.3, 22.3–100)	81.8 ± 21.1 (90.4, 7.3–100)
**PCR-NAWM**	71.6 ± 26.4 (78.7, 14.8–100)	52.6 ± 29.2 (48.8, 7.1–99.8)

Completers-only sample: N = 41. Values represent: mean % ± sd (median, min-max) of the tract-based regional NAWM relative to ROI volume.

Table 6 shows the results of mixed model analysis between mobility and regional DTI changes within the NAWM of the sub-regions of the corpus callosum (CC) and corona radiata (CR). Changes in the GCC-NAWM-RD, GCC-NAWM-FA, and BCC-NAWM-RD were significantly associated with changes in walk time (p-value: 0.01, 0.02, 0.04, respectively). The only regional variable that showed significant association with Tinetti mobililty assessment was SCC-RD (p = 0.04). After correction for multiple comparisons the above correlations between walk time and both GCC-NAWM-FA and GCC-NAWM-RD remained significant while the one involving BCC-NAWM-RD could be considered a likely false positive (type I error). The observed association between SCC-NAWM-RD and Tinetti is also above the FDR-corrected p-value threshold for a top rank value (i.e. p = 0.017) and therefore at risk of being a false positive.

**Table 6:** Longitudinal analysis of diffusion indices of regional NAWM and mobility: Linear mixed model estimates and standard errors.

DTI Data	Walk Time*		Tinetti Mobility	
	Beta (SE)	P-Value	Beta (SE)	P-Value
**FA**				
BCC-NAWM	-0.92 (0.5)	0.08	4.69 (5.88)	0.43
GCC-NAWM	-1.03 (0.43)	**0.02**	4.59 (5.13)	0.38
SCC-NAWM	-0.39 (0.62)	0.54	11.92 (7.20)	0.11
ACR-NAWM	0.01 (0.64)	0.99	-12.25 (7.33)	0.10
PCR-NAWM	0.59 (0.44)	0.18	-8.1 (5.07)	0.12
SCR-NAWM	0.47 (0.52)	0.38	-10.09 (5.91)	0.10
**AD**				
BCC-NAWM	0.09 (0.41)	0.82	-0.41 (4.81)	0.93
GCC-NAWM	0.08 (0.23)	0.72	0.13 (2.70)	0.96
SCC-NAWM	0.03 (0.25)	0.90	-4.27 (2.92)	0.15
ACR-NAWM	-0.08 (0.44)	0.85	3.27 (5.14)	0.53
PCR-NAWM	-0.02 (0.24)	0.94	-0.94 (2.88)	0.75
SCR-NAWM	0.36 (0.35)	0.30	-5.67 (3.99)	0.16
**RD**				
BCC-NAWM	0.75 (0.36)	*0*.*04*	-3.58 (4.25)	0.41
GCC-NAWM	0.83 (0.31)	**0.01**	-3.47 (3.65)	0.35
SCC-NAWM	0.37 (0.42)	0.38	-10.34 (4.85)	*0*.*04*
ACR-NAWM	-0.01 (0.44)	0.99	7.83 (5.02)	0.13
PCR-NAWM	-0.36 (0.35)	0.31	5.15 (4.11)	0.22
SCR-NAWM	-0.28 (0.61)	0.65	9.04 (6.94)	0.20

* Values were log-transformed (log_10_).

AD: axial diffusivity; FA: fractional anisotropy; RD: radial diffusivity; NAWM: normal-appearing white matter; GCC, BCC, SCC: genu, body and splenium of corpus callosum. ACR SCR PCR: anterior, superior and posterior corona radiata. All models controlled for age, gender, BMI, time point and baseline WMHv (log_10_). The values of p ≤ 0.05 but considered not significant after correction are indicated in italic (BCC-NAWM-RD and SCC-NAWM-RD). The p-values that remained significant after correction for multiple comparisons are highlighted in bold (GCC-NAWM-FA and GCC-NAWM-RD).

#### Mobility and DTI changes: Voxel-based whole-brain analysis

The exploratory whole-brain voxel-based analysis was performed to complement the analyses on global supratentorial WM and selected white matter tracts, and to provide additional regional information regarding microstructural abnormalities in different brain structures potentially associated with mobility impairment.

**Fig 6:**
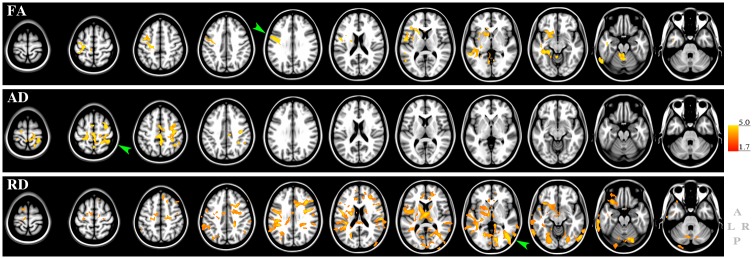
Voxel-wise analysis of DTI and Tinetti mobility. The figure shows the results of the voxel-wise general linear model statistics to assess association between changes in Tinetti total mobility score and changes in brain DTI indices. Top: FA; middle: AD, bottom: RD. The red-to-yellow colors indicate the T-values in areas of significant association. The bar on the right represents min and max of the T-value (1.7 to 5.0) for a one-tailed threshold of p<0.05 at the voxel level. The higher the T the more significant is the association. Results are overlaid on the Montreal Neurological Image brain template for anatomical reference. Green arrowheads indicate areas with highest significance. Image orientation is shown at bottom right: anterior (A), posterior (P), left (L) and right (R).

We observed no significant associations between walk time changes and FA, AD and RD changes. We found a significant but relatively modest association between changes in Tinetti score and FA, AD and RD changes (Fig 6). The FA changes showed a positive association with Tinetti score, and were localized mainly in the white matter of the left hemisphere. The pre-central gyrus (primary motor area) showed the most significant association with changes in Tinetti score (Fig 6, green arrowhead). Other significant clusters were located within the nucleus accumbens, post-central gyrus, para-central, caudal-middle-frontal, anterior limb of the internal capsule, anterior corona radiata, the genu of corpus callosum (GCC), the external capsule, left inferior cerebellar peduncle, and cerebellum.

Changes in the AD index showed positive association with Tinetti score changes predominantly in the superior part of the right hemisphere, above the centrum semiovale. The cluster with the highest significant association was located within the supra-marginal gyrus (Fig 6, green arrowhead). Other areas showing significant association with Tinetti score changes were the pre- and post-central gyri, superior- and inferior-parietal gyri, and superior longitudinal fasciculus.

Changes in RD showed more widespread, bilateral spatial pattern of negative association with changes in Tinetti score. The most significant association localized in the right occipital lobe (green arrowhead). Other brain areas showing significant association with Tinetti score changes were located in the supra-marginal and lingual white matter and cortex (right hemisphere), middle frontal white matter (left hemisphere). Additional associations were observed in the pre-central and superior-frontal white matter (bilaterally), corona radiata, corpus callosum, inferior-parietal white matter (left hemisphere), post-central white matter (right hemisphere), internal capsule (left hemisphere), thalamus (bilaterally), and cerebellum (bilaterally).

In summary, Tinetti mobility score changes showed the most prominent association with diffusion changes in subcortical white matter areas, i.e. left pre-central gyrus (FA), right superior-parietal (AD), and right lateral occipital (RD) white matter.

## Discussion

Our study comparatively assessed changes in diffusion indices of microstructural integrity within brain NAWM and WMH, and the association of NAWM measures with mobility changes. The expected difference in DTI indices between NAWM and WMH reflects the relative higher amount of free water in the latter WM compartment, and is consistent with previous findings supporting the ability of DTI to detect white matter damage in older persons [41]. The results of our longitudinal analysis are novel and may provide insight regarding possible processes underlying WMH lesion progression.

The 4-year reduction in water diffusion anisotropy, i.e. FA, within the cerebral NAWM is notable given the relatively short interval between observations. The decrease of NAWM-FA is consistent with the observed reduction of AD and increase of RD. Lower FA with higher RD values have been previously reported in a longitudinal 2-year study of 8 older subjects (mean age = 74 years, range = 65–81 years) using regional analysis of the corpus callosum [42]. High RD is generally considered a result of demyelination [43]. Low AD has been observed in mice following acute axonal injury [43] and in humans immediately after callosotomy during a period of axonal dissolution with relative preservation of myelin [44]. Thus the pattern we observed in our study seems consistent with axonal as well as myelin deterioration in the brain NAWM over time.

The global profile of the water diffusion indices within the WMH, characterized by lower FA and higher AD and RD, is in striking contrast with that of the NAWM. We interpret the low anisotropy and corresponding high values of both RD and AD, as evidence of microstructural tissue disorganization within areas of WMH. We can only speculate on the underlying processes. The expected higher water movement both along and perpendicular to the neural tracts within areas of WMH is consistent with loss of white matter microarchitecture [45–47] and it may involve both axonal and myelin loss [48, 49], tissue rarefaction and gliosis [50]. Clearing of cellular debris in damaged areas by microglia [51] may also contribute to the observed increase in WMH-AD after four years. Our results appear consistent with published histopathology and DTI studies. Areas of WMH are heterogeneous in size, location, and texture, and likely represent a variety of tissue abnormalities at the histological level [50, 52, 53]. Although ischemia is considered one probable underlying cause of deep and confluent WMH, the etiology of the tissue injury causing WMH remains unclear and may also vary with WMH location. Interestingly, the results of our longitudinal analysis within WMH showed evolution of the diffusion signal over time, which may reflect different degrees and substrates of white matter damage. A detailed analysis of WMH heterogeneity was beyond the scope of this work, leaving the relative contribution of WMH size, location, and texture to be determined in future studies.

In cross-sectional analyses on our relatively large baseline sample data, larger WMH burden was associated with lower NAWM-FA and both higher NAWM-AD and NAWM-RD. This observation is consistent with findings from other studies [21, 54–56]. Our results and those from others [55–57] taken together support a link between WMHv severity and degraded supratentorial NAWM microstructure. It is possible that white matter lesions disrupt not only the local tissue structure within the affected areas but also the related broader neural circuit [57] through anterograde (Wallerian) [58] and retrograde degeneration along the neural tracts. Another explanation is based on shared risk factors, e.g. age, hypertension and/or metabolic syndrome, that underlie the processes leading to both WMH and the microstructural changes in the NAWM. These processes co-evolve over time with some of the DTI changes preceeding visible WMH seen in conventional T2-weighted MRI [59, 60]. Since alteration in blood brain barrier permeability of NAWM in subjects with small vessel disease has also been reported [61], it is possible that areas of WMH represent the "tip of the iceberg" of more widespread WM abnormalities linked to defective blood brain barrier permeability [61–63].

In our analysis of the link between global white matter hyperintensity burden (WMHv) and DTI-based indices we observed that WMHv correlated relatively more strongly to WMH-AD than to NAWM-AD. This could be due to gliosis-related processes occurring within WMH areas. We speculate that the relatively higher AD (parallel diffusion) in the WMH compartment compared to NAWM might relate to microglial clearing of axonal cellular debris within older parts of the WMHv lesion [51]. Such process might also underly the longitudinal observation of an WMH-AD increase, which in turn explains the small but significant increase in WMH-FA seen at four years. Regional increases in FA have been described within intersecting fiber tracts, e.g. in the rostral pons. When secondary degeneration affects only one of the intersecting tracts the principal eigenvector (i.e. AD) of the remaining intact fibers would result in increased FA [64]. While occurrence of the latter is conceivable, it would be a small contribution as we examined global WMH diffusion. Focused studies are required to further define the processes underlying this observation.

Besides the possible interpretations regarding the substrates of WMH and NAWM damage, the observed association between WMH burden and loss of microstructural integrity within the NAMW, together with the differences in DTI indices between NAWM and WMH, supports the notion that diffusion NAWM abnormalities represent areas of tissue at risk for evolution to macroscopic WM damage [54]. These NAWM diffusion indices may be used to stratify subjects based on their risk of small vessel disease progression, and to plan interventions aimed at preventing WMH accrual, such as intensive control of blood pressure [65].

In relation to the functional impact, we consider low microstructural integrity within NAWM a form of white matter damage potentially additive to that linked to visible WMH. The findings from this longitudinal study indicate that the global reduction in microstructural integrity of the cerebral NAWM correlates weakly with mobility decline in elderly individuals. However, integrity reduction in the NAWM of the corpus callosum, particularly within the genu (GCC), is more strongly associated with mobility deterioration.

A previous cross-sectional study demonstrated DTI evidence of damage in NAWM of anterior corpus callosum and centrum semiovale, which correlated with measures of mobility [13]. Another cross-sectional study showed FA evidence of white matter damage in some of the same areas correlated with poorer Tinetti Scores [28]. Our regional study provides for the first time longitudinal assessment of DTI variables of NAWM of the corpus callosum and corona radiata. The critical roles of functioning ascending/descending pathways and inter-hemispheric connections in motor, somatosensory and visual integration explain the effects on mobility of microvascular damage within the corpus callosum and corona radiata. This explanation seems consistent also with the results of a recent five-year study on gait decline [29].

The longitudinal relationship between mobility and FA/RD within the NAWM of CC sub-regions adds to previously reported association between callosal WMH volume and impaired mobility [1, 30]. While study design and sample size differences make the comparison difficult, the MRI data suggest at least two complementary causes for the decline in mobility function. In addition to the known association with WMH burden, the degree of regional microstructural abnormalities within the NAWM, particularly in the corpus callosum, may also contribute to the decline in mobility function with a mechanism involving reduced connectivity caused by demyelination and axonal damage. This is specifically supported by the analyses adjusted for WMH burden, which showed a relatively mild but significant association between NAWM abnormalities and mobility impairment. Our finding of a stronger involvement of the genu compared to other parts of the corpus callosum is consistent with previous reports [13, 15].

The purpose of the brain wide voxel-based approach was to complement the ROI approach and to verify that we did not miss regions potentially involved in the development of mobility impairment, either in the white or gray matter. Results of the longitudinal analysis suggest a weak (Tinetti) or no (walk time) association between mobility and microstructural changes, with no evidence of a particular anatomical pattern. FA, AD, and RD identified different spatial patterns of microstructural abnormalities associated with changes in Tinetti score, including primary motor, somatosensory associative, and visual subcortical areas. In addition to normal-appearing and WMH areas, these regions involve cortical and subcortical gray matter structures distinct from the deep WM ROIs we selected for our regional NAWM analysis (i.e., the corona radiata and corpus callosum), and distant from the periventricular areas where WMHs mostly occurred. Given the observed relatively weak association, the contributions of these additional areas to mobility changes may be relatively modest and therefore they may need to be investigated in future larger studies.

We acknowledge a number of limitations of this study. There are drawbacks in both the whole-brain voxel-based and WM sub-compartmental analyses. The former is a group analysis method with spatial normalization to a template brain with low spatial resolution output maps, and cannot distinguish between NAWM and WMH at the level of the individual brain. The NAWM and WMH compartmental approach using average measures does not allow anatomical localization of association. Our regional tract-based approach attempted to overcome some of these obstacles. We restricted the regional analysis to two supratentorial WM tracts due to limited statistical power provided by our relatively small sample of participants with longitudinal data. We also acknowledge the limitations of using FDR instead of a family-wise method to correct for multiple comparisons, which favored a relatively high sensitivity to avoid missing potentially relevant associations at a cost of lower specificity.

In conclusion, this is the first longitudinal report of comparative microstructural changes in the NAWM and WMH and their association with measures of mobility in community-dwelling older individuals. Although significant, our findings indicate a relatively mild effect of loss of NAWM microstructural integrity on the development of mobility impairment, thus obliging confirmation in larger studies. Finally, the observed link between decreasing microstructural integrity in NAWM and increasing WMHv burden provides added urgency to efforts to better understand the association of vascular disease risk factors with brain microvascular abnormalities and to develop effective treatment to prevent clinically relevant white matter damage [65].

## Supporting information

S1 Table Correlation of mobility with cerebral NAWM DTI indices. Baseline sample, N = 86. Values indicate rho from Spearman correlation test. Values that remain significant after controlling for age and gender are indicated in bold. Significant values not highlighted in bold become not significant after controlling for age, gender and baseline-WMHv. WMHv: baseline volume of white matter hyperintensities; NAWM: normal-appearing cerebral white matter; AD: axial diffusivity; FA: fractional anisotropy; RD: radial diffusivity; n.s. = not significant (p>0.05).
(PDF)

S1 Scatterplot Completers-only sample, N = 41. Longitudinal changes from baseline (i.e. 0 years) to follow-up (i.e. 4 years) in FA (top), AD (middle, columns 1 and 2), RD (bottom) and WMHv (middle, column 3). NAWM: normal-appearing cerebral white matter; WMH: cerebral white matter hyperintensities; FA: fractional anisotropy (arbitrary units); AD: axial diffusivity (μm^2^/sec); RD: radial diffusivity (mean μm^2^/sec); WMHv: volume of white matter hyperintensities expressed as percent of intracranial volume.
(PDF)

S1 Data Data underlying the findings described in the manuscript. The file S1 Data contains the baseline and four years follow-up (4yrs) data for the subjects in the study. Measures are: age, gender, body mass index (BMI), level of education, mini-mental status exam (MMSE), walk time, Tinetti Total score, and white matter hyperintensity volume (WMHv).
(XLSX)

S2 Data Data underlying the findings described in the manuscript. The file S2 Data contains the global diffusion indices at baseline and at four year follow-up (4yrs). The indices are: mean axial diffusivity in cerebral normal appearing white matter (AD_NAWM) and white matter hyperintensities (AD_WMH), mean fractional anisotropy in cerebral normal appearing white matter (FA_NAWM) and white matter hyperintensities (FA_WMH), and mean radial diffusivity in cerebral normal appearing white matter (RD_NAWM) and white matter hyperintensities (RD_WMH).
(XLSX)

S3 Data Data underlying the findings described in the manuscript. The file S3 Data contains the regional diffusion indices at baseline and at four year follow-up (4yrs). The measures represent the mean axial diffusivity (AD), radial diffusivity (RD) and fractional anisotropy (FA) in the normal appearing white matter (NAWM) of the genu, body and splenium of the corpus callosum (GCC, BCC, SCC, respectively) and in the anterior, superior and posterior parts of the corona radiata (ACR, SCR, PCR, respectively).
(XLSX)

S4 Data Data underlying the findings described in the manuscript. The file S4 Data contains the regional percent of normal appearing white matter (NAWM) and white matter hyperintensity (WMH) in the genu, body and splenium of the corpus callosum (GCC, BCC, SCC, respectively) and in the anterior, superior and posterior parts of the corona radiata (ACR, SCR, PCR, respectively).
(XLSX)
