# Supplementary material for: Longitudinal microstructural changes of cerebral white matter and their association with mobility performance in older persons
Source: PLoS One. 2018 Mar 19;13(3):e0194051. doi: 10.1371/journal.pone.0194051 (PMC5858767; doi:10.1371/journal.pone.0194051)
Supplement: S1 Table — Baseline sample, N = 86. Values indicate rho from Spearman correlation test. Values that remain significant after controlling for age and gender are indicated in bold. Significant values not highlighted in bold become not significant after controlling for age, gender and baseline-WMHv. WMHv: baseline volume of white matter hyperintensities; NAWM: normal-appearing cerebral white matter; AD: axial diffusivity; FA: fractional anisotropy; RD: radial diffusivity; n.s. = not significant (p>0.05). (PDF) [file pone.0194051.s001.pdf]

S1 Table. Correlation of mobility with cerebral NAWM DTI indices

	**Walk Time rho (p)**	**Tinetti Mobility rho (p)**
NAWM-FA	-0.289 (0.007)	n.s.
NAWM-AD	n.s.	-0.213 (0.05)
NAWM-RD	0.303 (0.004)	n.s.
WMHv	**0.414 ($7*10^{-5}$)**	**-0.283 (0.008)**

Baseline sample, N=86. Values indicate rho from Spearman correlation test. Values that remain significant after controlling for age and gender are indicated in bold. Significant values not highlighted in bold become not significant after controlling for age, gender and baseline-WMHv. WMHv: baseline volume of white matter hyperintensities; NAWM: normal-appearing cerebral white matter; AD: axial diffusivity; FA: fractional anisotropy; RD: radial diffusivity; n.s. = not significant (p>0.05).
