# Supplementary figures and images for: Longitudinal microstructural changes of cerebral white matter and their association with mobility performance in older persons
Source: PLoS One. 2018 Mar 19;13(3):e0194051. doi: 10.1371/journal.pone.0194051 (PMC5858767; doi:10.1371/journal.pone.0194051)

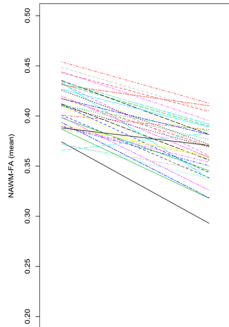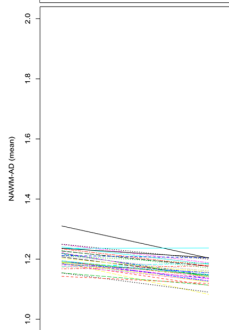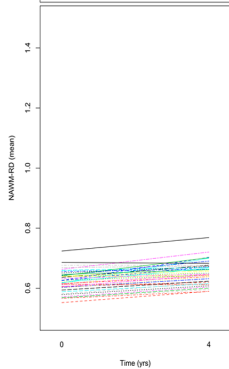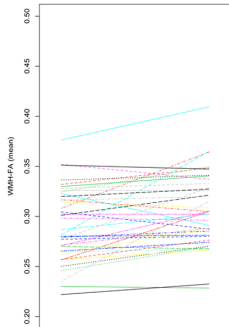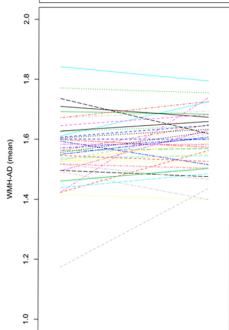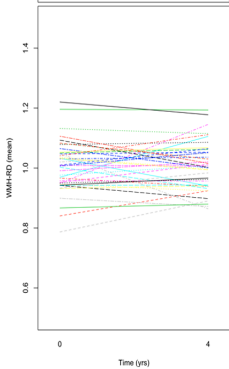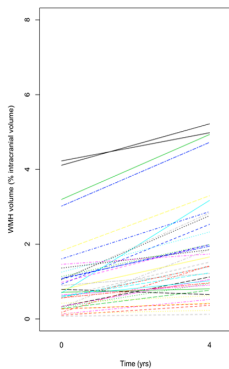

Supplement: S1 Scatterplot — Longitudinal changes from baseline (i.e. 0 years) to follow-up (i.e. 4 years) in FA (top), AD (middle, columns 1 and 2), RD (bottom) and WMHv (middle, column 3). NAWM: normal-appearing cerebral white matter; WMH: cerebral white matter hyperintensities; FA: fractional anisotropy (arbitrary units); AD: axial diffusivity (μm2/sec); RD: radial diffusivity (mean μm2/sec); WMHv: volume of white matter hyperintensities expressed as percent of intracranial volume. (PDF) [file pone.0194051.s002.pdf]
